# Neuro-Immune Axis in Trauma-Induced Heterotopic Ossification: Mechanisms and Therapeutic Implications

**DOI:** 10.3390/cells15090827

**Published:** 2026-05-01

**Authors:** Oluomachukwu Jennifer Agu, Clifford Pereira, Ishaan Gupta, Ashley Moran, Tahmineh Mokhtari

**Affiliations:** Department of Surgery, University of California Davis Medical Center, Sacramento, CA 95817, USA; ojagu@health.ucdavis.edu (O.J.A.); igupt@health.ucdavis.edu (I.G.); amoran3@health.ucdavis.edu (A.M.)

**Keywords:** heterotopic ossification, neural–immune axis, neural inflammation, blood–nerve barrier, neural crest-derived progenitor cells

## Abstract

**Highlights:**

**What are the main findings?**

- We review the neuro-immune axis in trauma-induced heterotopic ossification (tHO), which is a paradigm shift from the conventional “osteo-centric” view of heterotopic ossification.
- The neural–immune interactions in tHO are the earliest steps in the initiation of tHO and inform resident mesenchymal stem cells/progenitor cell fate.

**What are the implications of the main findings?**

- Early neural–immune interactions are attractive targets for novel therapeutics that could prevent and mitigate tHO prior to the clinically irreversible downstream BMP/Smad 1,5,8 osteogenesis pathway.

**Abstract:**

Trauma-induced heterotopic ossification (tHO) is characterized by aberrant ectopic bone formation in soft tissue following high-energy trauma, affecting >60% of combat-related amputees and >50% of major burn patients. Current prophylactic strategies (including NSAIDs, bisphosphonates, and low-dose radiation) lack mechanistic specificity, carry significant side effects, and surgical excision carries a 27% recurrence rate. This review reframes tHO pathogenesis through the neural–immune axis, arguing that ectopic bone formation is a downstream consequence of dysregulated neuroimmune signaling rather than a primary osteogenic event. Following trauma, nociceptor activation drives nociception-induced neural inflammation (NINI), releasing substance P (SP) and calcitonin gene-related peptide (CGRP), which disrupts the blood–nerve barrier, mobilizes neural crest-derived progenitor cells, and, alongside BMP-2/SMAD1/5/8 signaling and M1-polarized macrophage activation, establishes a permissive osteogenic microenvironment. A BMP-2/CGRP positive feedback loop sustains aberrant osteogenesis, converging on osteogenic transcription factors Runx2, SOX5/6/9, and Osterix. Dysregulated noncoding RNAs represent promising pre-radiographic biomarkers. This neural–immune framework motivates mechanism-based therapeutic strategies targeting CGRP (fremanezumab, erenumab), SP/NK1 signaling (aprepitant), and macrophage polarization (metformin, palovarotene, rapamycin), with multi-node combination approaches tailored to the temporal stages of tHO offering the most promise for precision prophylaxis.

## 1. Introduction

Heterotopic ossification is a debilitating condition characterized by aberrant formation of lamellar bone in soft tissue [1]. It arises secondary to severe trauma or rare genetic conditions like fibrodysplasia ossificans progressiva, Albright’s hereditary osteodystrophy and progressive osseous heteroplasia [2]. Trauma-induced heterotopic ossification (tHO) is more common and results from high-energy amputations (>60%), spinal cord injuries (20–30%), closed head injuries (10–20%), and major burns (>50%). Symptomatic tHO is found in 62.9% of combat-associated and 22.5% of civilian-associated amputations, and the incidence increases to 79.6% if the amputation is performed through the zone of injury [3,4,5,6,7,8,9,10]. In addition to trauma, tHO also occurs commonly after elective hip and knee arthroplasties. More than 50% of the 2.2 million patients undergoing elective hip and knee arthroplasties in the United States each year develop tHO, of which >10% are severe enough to warrant surgery due to joint stiffness and severe pain [11]. Heterotopic bone can cause severe pain due to compression of overlying skin, recurrent skin breakdown, recurrent infections, etc., often resulting in opioid dependence. It can also restrict joint movement, leading to gait impairment, prosthetic abandonment in amputees, and disrupt activities of daily living [12,13].

Despite the fact that tHO was first recognized in the early 1800s, there is a paucity of effective prophylactic or therapeutic strategies. Preventative measures for tHO are limited to non-steroidal anti-inflammatory drugs (NSAIDs), low-dose radiation, and bisphosphonates, all with significant side effects and dismal long-term efficacy [4,5,6]. Moreover, these prophylactic interventions are applied empirically, given the current lack of reliable biomarkers to identify patients at high risk, thus exposing patients to unnecessary interventions and avoidable harm. Surgical excision, reserved for intractable cases, is usually incomplete due to diffuse aberrant bone often encasing neurovascular structures, and has a 27% recurrence rate [14,15,16]. The lack of effective prophylaxis and treatments for tHO highlights the imperative need to further elucidate the mechanisms of tHO initiation, with the goal of developing novel therapeutic strategies. The role of the immune system (macrophages, mast cells, and pro-inflammatory cytokines) has been well established in tHO pathogenesis; however, emerging evidence suggests the critical role of peripheral nervous system during the initiation of ectopic bone formation [17,18]. While the classical inflammatory and BMP/SMAD paradigms have substantially advanced our understanding of tHO [19,20], they do not fully account for the upstream events that initiate and sustain aberrant osteogenesis, highlighting the need for an integrative framework that incorporates the role of neural activation in the earliest stages of tHO pathogenesis [21,22].

Recent advances in understanding the role of the peripheral nervous system and the immune system have emerged from the idea of their strong cross-interactions and their common emphasis on integration of external environmental stimuli and maintaining homeostasis [13]. In this review, we highlighted the neural–immune interactions underlying tHO pathogenesis, emphasizing the mechanistic crosstalk that initiates and sustains aberrant osteogenesis. We also highlight emerging therapeutic targets and promising research directions at these critical intersections. With this in mind, we will first review how inflammatory dysregulation, and neural inflammation in particular, contributes to the misdirected differentiation of progenitor cells down the osteogenic pathway.

## 2. Overview of tHO

tHO proceeds through endochondral ossification, recapitulating developmental cartilage-to-bone transition programs; however, the upstream molecular and neuroimmune signaling mechanisms that initiate and sustain this ectopic process remain incompletely defined. Increasing evidence implicates inflammation as a central contributor to tHO pathogenesis, particularly in the early post-traumatic phase [20]. Following injury, the inflammatory response not only initiates tissue repair processes but also interacts closely with nociceptive signaling pathways, giving rise to nociception and resultant neural inflammation [23]. A central mediator of linking tissue injury to neural activation is nerve growth factor (NGF), which is rapidly upregulated in damaged tissues by multiple local cell populations, including vascular smooth muscle cells, pericytes, inflammatory cells, and stromal elements [24,25]. NGF binds with high affinity to tropomyosin receptor kinase A (TrkA) expressed on nociceptive sensory neurons, initiating intracellular signaling cascades that enhance neuronal excitability [26].

One important downstream consequence is sensitization of ion channels responsible for detecting harmful stimuli, particularly members of the transient receptor potential (TRP) family such as transient receptor potential vanilloid-1 (TRPV1) [27]. TRP channels comprise a large superfamily of cation-permeable channels involved in sensory transduction. Within this group, the vanilloid subfamily (TRPV1–TRPV6) responds to thermal and chemical stimuli [26]. TRPV1 is the most extensively studied member and functions as a polymodal detector of tissue injury, activated by noxious heat, acidosis, inflammatory mediators, and capsaicin. Following trauma, activation of TRPV1 lowers the threshold for nociceptor firing, thereby producing hyperalgesia and persistent pain. In addition to responding to external stimuli, TRPV1 can be activated indirectly by cellular damage signals, linking structural injury to neural activation. The NGF-TrkA complex is subsequently endocytosed in the sensory axon and retrogradely transported to the dorsal root ganglion (DRG) cell body via signaling endosomes [28]. This retrograde trafficking enables sustained intracellular signaling that alters gene expression programs within nociceptors. Activation of mitogen-activated protein kinase pathways, including p38 MAPK, leads to increased transcription of pro-inflammatory mediators and further upregulation of TRPV1 expression. Consequently, injured sensory neurons become progressively more responsive to subsequent stimuli, amplifying nociceptive signaling over time [29].

**Figure 1:**
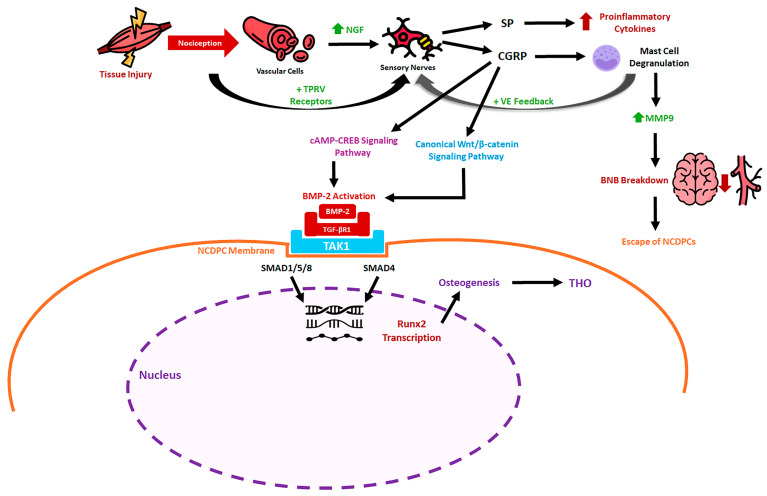
Mechanism linking nociception, neuroinflammation, and osteogenic differentiation in NCDPCs. Schematic illustration of signal transduction pathways involved in NINI and tHO. NINI occurs via the release of NGF from vascular pericytes and the ultimate release of SP and CGRP from sensory nerves within the injured tissue. NGF binds to TrkA on nociceptive sensory neurons and sensitizes TRPV1 channels, lowering the threshold for nociceptor firing and amplifying nociceptive signaling. This leads to degranulation of mast cells, the release of MMP9, and proinflammatory cytokines, leading to the breakdown of the blood–nerve barrier, and release of NCDPCs into the injured tissue. Parallel to this process, BMP-2 initiates the NCDPCs toward osteogenic differentiation. CGRP has direct osteogenic effects on osteoprogenitor cells/mesenchymal stem cells by activating BMP-2 via canonical Wnt/β-catenin signaling and CREB signaling. A positive feedback loop exists whereby BMP-2 further upregulates CGRP expression in sensory neurons, while CGRP in turn amplifies BMP-2 production, sustaining and escalating osteogenic signaling at the injury site. BMP-2 binds to TGF-βRI and activates TAK1, leading to phosphorylation of SMAD1/5/8, which binds to the co-activator SMAD4 and translocates to the nucleus to serve as a transcription factor for BMP-responsive genes critical in osteogenesis such as Runx2 and others. Collectively, these converging neuro-immune signals drive NCDPCs to escape damaged peripheral nerves, extravasate into injured soft tissue, and differentiate into osteoblasts, ultimately resulting in ectopic bone deposition and tHO. Arrow legend: ↑ green, upregulation; ↑ red, pro-inflammatory activation; ↓ red, downregulation or barrier disruption (e.g., BNB integrity loss).

In parallel with neuronal sensitization, NGF promotes structural remodeling of peripheral nerves. Injury-induced NGF gradients drive sprouting and ingrowth of TrkA-expressing sensory and sympathetic fibers into damaged tissues. Experimental studies have demonstrated that disruption of neural input—such as by surgical denervation—significantly impairs this axonal invasion and reduces heterotopic bone formation [9]. Transcriptomic analyses of denervated tissues reveal marked shifts in signaling pathways within mesenchymal progenitor populations, including decreased TGF-β signaling and increased fibroblast growth factor activity, changes that favor fibrotic repair over endochondral ossification. Consistent with these observations, genetic or pharmacologic interruption of NGF–TrkA or TRPV1 signaling attenuates tHO in animal models [9,18,30]. Together, these findings support the concept that sensory neurons provide instructive signals that guide aberrant skeletal differentiation following injury. Clinical observations align with these mechanistic insights. The likelihood of tHO formation correlates strongly with injury severity, with high-risk scenarios including extensive burns, traumatic brain injury (TBI), spinal cord injury, sepsis, and major fractures [31,32]. These conditions are associated with profound nociceptive activation and systemic inflammation, suggesting that excessive neural signaling contributes to pathological ossification [33]. This process has been conceptualized as nociception-induced neural inflammation (NINI), in which activated sensory neurons release bioactive neuropeptides that modulate immune responses and tissue remodeling [30] (See Figure 1).

Two key neuropeptides released from activated nociceptors are substance P (SP) and calcitonin gene-related peptide (CGRP). Both are stored in vesicles within peripheral nerve terminals and are rapidly released in response to depolarization, TRPV1 activation, or inflammatory signals [34,35]. SP, a member of the tachykinin family, exerts potent pro-inflammatory effects by stimulating macrophages, monocytes, and mast cells to produce cytokines and chemokines. It also promotes mast cell degranulation, leading to the release of proteases and histamine that increase vascular permeability and recruit additional immune cells. Elevated circulating levels of SP have been detected in conditions associated with heterotopic ossification, including severe trauma and fibrodysplasia ossificans progressiva. Experimental blockade of the SP receptor neurokinin-1 reduces ectopic bone formation, highlighting the importance of this signaling axis [30,36]. Beyond its immunomodulatory effects, SP can directly influence mesenchymal progenitor behavior. In vitro studies demonstrate that SP enhances migration of mesenchymal stem cells and can promote osteogenic differentiation, suggesting a mechanism by which neural signals recruit and activate progenitors at sites of injury. Increased SP levels observed after TBI may therefore contribute to the heightened risk of heterotopic ossification in these patients [37]. CGRP, a 37-amino-acid peptide derived from alternative splicing of the calcitonin gene, represents another major effector of sensory neuron activity [38]. The predominant isoform, α-CGRP, is synthesized primarily in DRG neurons and transported to peripheral terminals in an NGF-dependent manner and promotes vasodilation, mast cell degranulation, cytokine release, neovascularization, and influences the function of both osteoblasts and osteoclasts [39]. At the cellular level, CGRP signals through a receptor complex composed of calcitonin receptor-like receptor (CRLR) and receptor activity-modifying protein-1 (RAMP1). This receptor is widely expressed on stromal, immune, and skeletal progenitor cells. Activation triggers intracellular pathways such as cyclic AMP signaling and Wnt/β-catenin activation, both of which are associated with osteogenic differentiation. In in vivo models of tHO, CGRP has been shown to promote recruitment of inflammatory cells and to enhance osteogenic commitment of mesenchymal progenitor cells (MPCs) [40,41,42].

Bone morphogenetic proteins (BMPs) provide a crucial link between neural activation and osteogenic differentiation. Members of the BMP family, part of the broader TGF-β superfamily, are key regulators of skeletal development and repair. Among them, BMP-2 has emerged as a central driver of heterotopic ossification across diverse contexts. It is produced at the nodes of Ranvier in sensory nerves and upregulates the secretion of SP and CGRP in DRG in a dose-dependent manner [43]. BMP-2 not only acts directly on mesenchymal progenitors but also influences neural signaling. It can increase production and release of SP and CGRP from sensory neurons, thereby amplifying neurogenic inflammation. Conversely, tissue damage elevates local levels of both BMP-2 and TGF-β, which together direct progenitor cells toward chondrogenic and osteogenic differentiation through canonical SMAD-dependent and noncanonical MAPK pathways [44,45]. In the SMAD-dependent pathway, BMP2 binds the heterotetrameric receptor complexes consisting of BMP type I receptors (BMPRI) and BMP type II receptors (BMPRII), and once bound, BMPRII phosphorylates and activates BMPRI [46]. BMPRI then phosphorylates SMAD1/5/8 [47,48]. SMAD1/5/8 binds the co-activator SMAD4, and this complex is translocated into the nucleus, acting as a transcription factor for Runx2, SOX5/6/9, and Osterix (Osx), leading to osteogenesis [44,45]. In the SMAD-independent pathway, BMP2 induces phosphorylation of protein kinase D in a protein kinase C-independent manner; protein kinase D subsequently phosphorylates p38 and promotes Runx2 phosphorylation [49]. Thus, both signaling cascades converge on transcription factors such as and Osx, which govern osteoblast lineage commitment. Additionally, BMP2 upregulates osteogenic differentiation by CGRP, which in turn upregulates BMP2 expression via a positive feedback mechanism in tHO [50]. Alongside neurogenic signaling, during the initial phase, infiltration and accumulation of monocytes and macrophages at the injury site, combined with increased metabolic activity and vascular damage, reduce oxygen availability in the inflamed region [51]. The resulting hypoxia activates inflammatory signaling cascades that further amplify and sustain the inflammatory response [51]. Persistent inflammatory and hypoxic cues within tHO lesions further promote the osteochondral differentiation of mesenchymal progenitors [52]. Chronic inflammation and hypoxia also modulate the expression of HIF-1α and HIF-1β, which in turn enhance VEGF-mediated angiogenesis in MSCs and potentiate BMP signaling, thereby driving both chondrogenic and osteogenic differentiation [53]. Collectively, these interconnected pathways drive endochondral ossification at ectopic sites.

**Figure 2:**
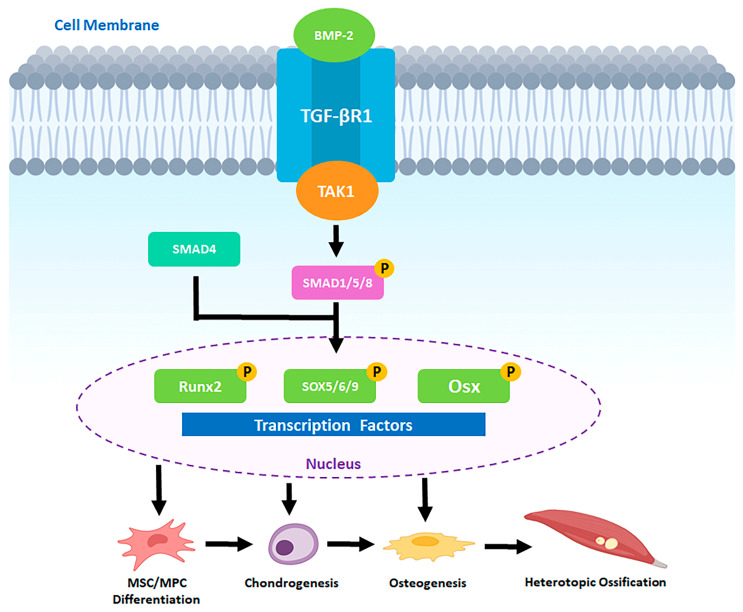
BMP-2 canonical signaling pathway in MSC/MPC osteogenic differentiation leading to tHO. Schematic illustration of the BMP-2 canonical SMAD-dependent signaling cascade driving MSC/MPC differentiation toward heterotopic bone formation. BMP-2 binds TGFβR1 at the cell membrane and activates TAK1, leading to phosphorylation of SMAD1/5/8. Phosphorylated SMAD1/5/8 forms a complex with the co-activator SMAD4 and translocates to the nucleus, where it acts as a transcription factor for Runx2, SOX5/6/9, and Osx. Activation of these transcription factors drives sequential MSC/MPC differentiation through chondrogenesis and osteogenesis, ultimately culminating in ectopic bone deposition characteristic of tHO. Phosphorylation events (denoted by P) at SMAD1/5/8, Runx2, SOX5/6/9, and Osx are essential for propagation of osteogenic signals from the cell membrane to the nucleus.

Neurogenic inflammation also recruits mast cells, which accumulate rapidly at sites of BMP-driven tissue injury. Degranulation of mast cells releases proteases and other mediators that remodel the extracellular matrix and facilitate nerve sprouting into damaged tissue. This remodeling appears to create a permissive microenvironment for heterotopic bone formation. Experimental stabilization of mast cells or depletion of macrophages significantly reduces ectopic ossification, demonstrating the importance of immune–neural interactions [9,26,54,55,56,57]. The walls of endoneurium of peripheral nerve axons contain the blood–nerve barrier (BNB), a single layer of endothelial cells comprised of tight junction molecules like occludin, zona occludens, and claudins [58]. The BNB restricts the entry of large molecules from blood vessels into nerves while allowing for easy exchange of gas and small molecules like the blood–brain barrier [59]. Mast cell degranulation caused by neuroinflammation releases matrix metalloproteinase-9 (MMP9) and histamine and activates perineural fibroblasts [60,61]. MMP9 and histamine decrease tight junction molecules, while perineural fibroblasts express Human Natural Killer-1 (HNK1), collectively increasing BNB’s permeability [55,56]. This BNB disruption allows BMP2 to enter the endoneurium and activate neural crest-derived progenitor cells (NCDPCs), which exhibit BMP2-SMAD1/5/8 signaling to trigger tHO [62]. Additionally, NCDPCs express *Osterix* (osteogenic transcription factor), then exit damaged nerves and transiently enter circulation [63]. At injured tissue, they extravasate and differentiate into osteoblasts that directly drive ectopic bone formation in tHO [62,63]. Collectively, these processes suggest that tHO represents a misdirected neurogenic repair response driven by aberrant neurovascular and stem cell signaling (See Figure 2).

## 3. Neural–Immune Axis (NIA)

The nervous and immune systems function as parallel surveillance networks that maintain physiological homeostasis and protect against environmental threats. Both systems colocalize at anatomical sites termed neuro-immune cell units (NICUs) [64,65]. These units can be found in several organs, including lymphoid and adipose tissue, skin, intestine, and lung [65]. In barrier tissues, such as the skin and gut, sensory nerve terminals are near macrophages and dendritic, mast, and innate lymphoid cells, enabling bidirectional communication that maintains physiological homeostasis [66,67]. Under steady-state conditions, neurons continuously shape immune cell phenotypes through their release of neurotransmitters and neuropeptides. For example, sympathetic innervation of lymphoid tissues influences immune cell positioning and hematopoiesis, while parasympathetic cholinergic signaling regulates immune cell activation thresholds, thereby maintaining an anti-inflammatory tone. In turn, emerging evidence further suggests that tissue-resident immune cells release cytokines to reciprocally shape baseline neuronal function and homeostatic circuits that control metabolism and other physiological processes [68,69].

During tissue homeostasis disruption, these same neuro-immune surveillance networks rapidly detect tissue injury and trigger coordinated inflammatory responses. Sensory neurons, such as nociceptors, act as rapid detectors of noxious mechanical, thermal, or chemical stimuli [70]. In parallel, immune cells continuously monitor their environment through pattern recognition receptors (PRRs), like toll-like receptors (TLRs), which recognize pathogen-associated molecular patterns (PAMPs) and detect tissue damage through endogenous damage-associated molecular patterns (DAMPs). Through shared molecular cues, including cytokines, growth factors, and neuropeptides, both systems can communicate bidirectionally and modulate each other’s tissue injury response [71]. Additionally, NICUs allow for rapid, localized neuroimmune coordination that can initiate responses within seconds to minutes of injury detection [70]. Lastly, convergent intracellular signaling pathways modulate ion channel activity, excitability, and inflammatory gene expression. As a result, both systems function as parallel, yet integrated, surveillance systems that can mount rapid responses during times of tissue injury. Following tissue injury, peripheral neurons and tissue-resident immune cells co-regulate an inflammatory signaling environment, within which immune-derived pathways play a central role in coordinating and amplifying local inflammation [72]. The interaction of PAMPs or DAMPs with different PRRs, such as TLRs or NOD-like receptors (NLRs), establishes the molecular framework of the acute inflammatory response due to the release of inflammatory signaling molecules. TNF, IL-6, and IL-1β drive early pro-inflammatory immune responses [73]. TNF and IL-1β promote endothelial activation, increasing vascular permeability and leukocyte recruitment [74], while IL-6 acts as an amplifying and integrative cytokine that contributes to sustained immune activation, acute-phase responses, and neuronal sensitivity modulation [75]. These cytokines do not act uniformly but instead form spatial gradients, with concentrations inversely proportional to their distance from the injury core in both muscle and vessel tissues. These gradients function to confine the inflammatory response, protecting non-injured tissue. Additionally, the local inflammatory response occurs in a temporally dependent manner. Primary inflammatory cytokines are released rapidly, within minutes of injury, while secondary mediators that amplify leukocyte recruitment are released at a later phase. For example, resident tissue macrophages accumulate at cardiac injury sites within 5 min, preceding neutrophil infiltration and monocyte/macrophage recruitment, both of which peak at 1 and 2–5 days, respectively. In parallel, mesenchymal stromal cells already present in the tissue rapidly respond to inflammatory signals. By day 1 post-injury, they express immunomodulatory transcripts, including chemokines, in response to, for example, IL-1β, TNF-α, and oncostatin M (OSM), a member of the IL-6 cytokine family, thereby contributing to neutrophil and macrophage recruitment. Notably, OSM has been identified as a key driver of neurogenic heterotopic ossification, as it promotes the osteogenic differentiation of MSCs [76,77].

Importantly, immune-derived signals do not act solely on immune cells but also modulate neuronal excitability. Peripheral sensory neurons, including nociceptors, embedded in injured tissues, express receptors for inflammatory cytokines, lipid mediators, and growth factors [78]. These immune-derived signals regulate neuronal excitability and activation thresholds, thus linking the early inflammatory immune response to neurosensitization [79]. While immune-derived signaling pathways regulate the magnitude, spatial distribution, and duration of inflammation, they simultaneously shape the neural response. This parallel, reciprocal inflammatory framework provides one part of the story in which neurogenic mechanisms further trigger and modulate responses to tissue injury [80,81].

Peripheral sensory neurons, located in NICUs, are positioned to detect tissue damage, respond to immune-derived inflammatory signals, and regulate local immune responses. Peripheral sensory neurons, specifically nociceptors, are specialized primary afferent neurons with cell bodies located in DRG and trigeminal ganglia that extend long peripheral projections terminating in the peripheral tissues as free nerve endings [82,83]. These neurons are positioned at barrier surfaces, enabling nociceptors to act as an early surveillance system, detecting tissue disruption and translating environmental signals into neural activity [84,85,86]. Upon activation, nociceptors trigger downstream signaling that extends beyond their sensory perception and actively alters the local tissue environment through neurogenic inflammation [87,88]. Although sensory neuron PRRs utilize canonical TLR signaling that drives transcriptional reprogramming, they also couple directly to ion channels to enable rapid modulation of neuronal excitability.

Nociceptors express multiple PRRs, including TLRs 2, 3, 4, 7, and 8, allowing for the detection of tissue injury [88,89,90,91]. These receptors enable direct sensing of DAMPs such as HMGB1 or miRNAs released by dying host cells, as well as PAMPs such as lipopolysaccharide or flagellin [92,93]. Unlike immune cells, which rely on slow transcriptional changes to mount a response, nociceptors can couple innate immune sensing to ion channels. TLR signaling can be linked to transient receptor potential channels such as TRPV1 and TRPA1, allowing nociceptors to modulate their neuronal excitability within seconds to minutes following injury detection [89,93,94]. Additionally, nociceptors express receptors for immune-derived cytokines and lipid mediators, including TNF-α, IL-6, and IL-1β receptors for PGE_2_ [95,96,97]. These receptors can then modulate voltage-gated sodium channels, lower activation thresholds, and promote nociceptor sensitization [98]. Through these mechanisms, inflammatory signals generated by immune cells directly influence neuronal sensitization, reinforcing bidirectional communication between the immune and nervous systems [99]. Neurogenic inflammation represents one component of continuous bidirectional neuroimmune communication that occurs during times of tissue injury. Activated nociceptors utilize efferent signaling to alter the local tissue environment through a process called neurogenic inflammation [87]. Signaling from nociceptors’ peripheral terminals triggers the release of neuropeptides, primarily SP and CGRP [92]. SP increases vascular permeability and promotes edema, while CGRP acts as a potent vasodilator, enhancing local blood flow [100]. Additionally, these neuropeptides act on resident and recruited immune cells. For example, nociceptor-derived signals can induce IL-23 production by dermal dendritic cells, which subsequently promotes IL-17A production by T cells and contributes to psoriatic skin inflammation [101,102]. Together, these nociceptor-driven mechanisms illustrate sensory neurons as active regulators of tissue inflammation, setting the stage for cell type-specific neuroimmune interactions.

Distinct immune cells at NICUs exhibit differences in receptor expression, functional roles, and signaling kinetics. Mast cells mount rapid responses to neural triggers due to their close anatomical association with peripheral nerve endings [103]. TRPV1-expressing nociceptors release the neuropeptide SP, which then binds to receptors on mast cells, triggering rapid degranulation with the release of histamine, TNF, and leukotriene B4 [104]. Beyond mast cell activation, sensory and autonomic neural signals also regulate neutrophil function [105]. Neutrophils can amplify and sustain the immune response. However, neural signaling can act as a suppressive regulator of neutrophil function. During invasive bacterial infections (e.g., *S. pyogenes* or *S. aureus*), nociceptors release CGRP, inhibiting neutrophil recruitment to infection sites and reducing phagocytic killing in certain tissue environments [106]. Additionally, macrophages play a prominent role as central sensors within the neuro-immune axis. Although macrophages are not exclusively tissue-resident, they are frequently found in close spatial association with peripheral nerve fibers at sites of inflammation and damage [107]. Their proximity to sites of damage allows for communication that influences their functional state. For example, as nearby sympathetic nerve terminals release norepinephrine, macrophage receptors bind the neurotransmitter, thus triggering an intracellular downstream cascade that increases cAMP and PKA while inhibiting the pro-inflammatory transcription factor NF-κB [108]. Through this mechanism, neural input modulates macrophage inflammatory tone and can contribute to tissue protection and resolution during injury and repair

## 4. Neural–Immune Communication

Neural regulation of immune function is primarily mediated by neuropeptides and neurotransmitters, which together exert context-dependent effects. In terms of neuropeptides, SP, CGRP, galanin, and somatostatin play a prominent role. As previously established, SP increases vascular permeability and triggers mast cell degranulation, thereby promoting neurogenic inflammation [109,110]. In contrast, CGRP often functions as a negative feedback signal. Upon its release, CGRP suppresses neutrophil recruitment and inhibits phagocytic killing, thus attenuating the innate immune response to prevent harmful overactivation [111]. Lastly, galanin and somatostatin suppress lymphadenopathy as well as inflammation during bacterial infection [112,113,114,115]. In addition to neuropeptides, neurotransmitters, including acetylcholine and catecholamines, act as regulators of immune function. Acetylcholine (ACh) is a key mediator of immune activation suppression. ACh functions as a negative feedback signal by binding nicotinic acetylcholine receptors on immune cells, thus reducing the production of pro-inflammatory cytokines [116]. Catecholamines, including norepinephrine and epinephrine, interact with beta-adrenergic receptors that are expressed on several immune cells. Upon immune-cell receptor binding, catecholamines trigger a downstream signaling cascade that increases intracellular cAMP/PKA activity and attenuates pro-inflammatory cytokine production [117]. Importantly, neural signals impact the immune system in a context-dependent manner. Neural signals (e.g., CGRP or catecholamines) that initially promote immune activation to support host defense can later shift toward inhibitory and/or suppressive roles to limit tissue damage [70]. These opposing effects are dependent on the inflammatory stage, cell type, and local conditions.

Immune-to-neural communication includes rapid sensitization during acute inflammation and transcriptional remodeling during chronic pain. Cytokines mediate immune regulation of neurons and drive feedback loops that amplify immune activation. Neurons can sense cytokines released by immune cells. Mediators such as NGF bind peripheral nerve terminals, triggering retrograde transport to the neuronal cell body and inducing sustained transcriptional and functional changes [118]. As a result, inflammation sensitizes neurons. Cytokines, prostaglandins, and lipid mediators reduce neuronal activation thresholds, thus sensitizing neurons and resulting in hyperalgesia and allodynia [96,119]. Additionally, inflammatory signaling pathways (MAPK, PKA, PKC) promote phosphorylation and functional modulation of ion channels such as TRPV1, TRPA1, and voltage-gated sodium channels (Nav1.7, Nav1.8, Nav1.9) [120,121,122]. This results in an amplification of neuronal excitability and an increase in pain signaling. Consequently, immune-derived signaling can induce lasting changes in neuronal sensitivity and gene expression, opening the pathway for inflammation to transition from acute and protective to chronic and pathological. This process is illustrated by chronic inflammation, which induces long-term upregulation of ion channels and neuropeptides within the DRG [123,124]. This is a feedback loop between immune activation and neural activity, where immune-derived cytokines sensitize neurons, increasing neuropeptide release, which in turn amplifies immune activation [109]. However, when this feedback loop becomes dysregulated, continuously activating the nervous system, tissues become predisposed to pathological inflammation. Thus, sustained or dysregulated neurogenic inflammation can shift neural–immune signaling from a reparative to a pathogenic program. Persistent neural activation disrupts normal resolution pathways of inflammation and tissue repair, since inflammatory tone, stromal responses, and tissue organization are altered [125]. Aberrant coordination between neural and immune systems promotes maladaptive tissue remodeling, thus providing a framework for understanding disease-specific dysregulation, including heterotopic ossification.

## 5. Neural–Immune Axis in tHO

tHO is traditionally conceptualized as an aberrant osteogenic process arising from severe trauma, and its pathogenesis involves dysregulated mesenchymal differentiation and excessive osteoinductive signaling [126]. Within this framework, ectopic bone formation is viewed as a localized failure of bone regulatory mechanisms that operates outside the skeletal compartment [126,127]. However, accumulating mechanistic evidence summarized in this review suggests a fundamental rethinking of tHO pathogenesis, though definitive clinical validation remains ongoing. In the aforementioned biochemical pathways, traumatic injury activates peripheral sensory and autonomic nerves alongside immune surveillance pathways, leading to neurogenic inflammation characterized by nociceptor sensitization, neuropeptide release, and immune cell activation [30,128]. These neural-derived signals amplify local inflammatory and vascular responses and establish a permissive microenvironment in which osteoinductive cues such as BMP signaling act on recruited progenitor cells [50,62,63]. Within our proposed framework, ectopic bone formation in tHO may not be the initiating pathological event, but rather a downstream consequence of coordinated and sustained neural–immune signaling. Osteoinductive cues act within a permissive microenvironment established by neural and immune activation, directing recruited progenitor cells toward an osteogenic fate rather than alternative regenerative outcomes [69,129,130]. Importantly, this perspective emphasizes that neural–immune pathways involved in normal wound healing and tissue repair are not inherently pathological; instead, they become maladaptive when their activation is prolonged or improperly resolved. Reframing tHO as a disorder of dysregulated neural–immune repair response provides a conceptual foundation for understanding disease progression and motivates further examination of how persistent signaling and failed resolution contribute to heterotopic bone formation.

It is important to distinguish what the neuro-immune framework adds beyond, and where it overlaps with, the classical BMP-driven and inflammatory models of tHO. The traditional inflammatory paradigm correctly identifies macrophage polarization, cytokine release, and immune cell recruitment as essential contributors to tHO [131,132]; the neuro-immune model extends this by demonstrating that these immune events are themselves orchestrated, at least in part, by nociceptor-derived neuropeptides such as SP and CGRP, which precede and amplify the macrophage response [13,131]. Similarly, the BMP/SMAD paradigm accurately describes the downstream osteogenic differentiation of mesenchymal progenitors [20]; however, it does not fully account for what initiates BMP-2 upregulation or why progenitor cells are mobilized specifically to sites of neural injury. The neuro-immune framework addresses this gap by positioning BMP-2 not solely as an osteoinductive effector but also as a neural activation signal capable of triggering CGRP and SP release from sensory neurons, thereby forming a positive feedback loop that sustains aberrant osteogenesis [133]. What is genuinely novel in the neuro-immune model, therefore, is the identification of neural activation and BNB disruption as the earliest upstream events in tHO pathogenesis; events that precede both the inflammatory surge and the BMP-driven osteogenic cascade, and that represent more tractable targets for early prophylactic intervention [134].

Although nociceptors have been well implicated in initiating and promoting tHO, emerging evidence suggests that tHO may arise from a broader, more complex pathway within the neuroimmune ecosystem. This pathway sits at the convergence of multiple neural, immune, and barrier-associated processes whose dysregulation leads to the osteogenic misallocation seen in tHO [30,135,136]. Emerging research suggests that the peripheral nerve itself may function as a reservoir of latent osteogenic potential. Peripheral nerves contain multiple progenitor populations capable of osteogenic differentiation [17,62,137]. These stem cells express Nanog and Klf4 and acquire osteoblast markers, including osterix, following BMP2 exposure. These findings suggest that osteogenic potential in tHO may not be induced de novo within injured muscle but rather may reflect an inherent characteristic of the peripheral nerve, a concept that warrants further in vivo investigation. Additionally, emerging evidence suggests that BMP2 may activate sensory nerves, potentially inducing neurogenic inflammation, nerve remodeling, and the release of osteogenic progenitors, though the precise sequence of these events remains to be fully established. While the precise sequence of these events is not completely understood, these observations reposition BMP not only as an osteoinductive cue but also as a neural activation signal [138]. Collectively, these observations support the concept that the peripheral nerve may act as an active upstream modulator of osteogenesis in tHO, reframing ectopic bone formation as a problem of progenitor misallocation rather than local mesenchymal induction.

Once neural activation has occurred, additional nerve-resident cell types may shape how this osteogenic potential is realized. Schwann cells have emerged as potential contributors to tHO pathogenesis, although their precise role remains incompletely defined [139,140]. Beyond their classical role in axonal support and repair, Schwann cells modulate immune responses and tissue remodeling within peripheral nerves. In vitro studies suggest that Schwann cells may promote the secretion of macrophage migration inhibitory factor (MIF), which activates downstream CD74/FOXO1 signaling and consequently promotes osteogenic differentiation of preosteoblasts, though whether this mechanism operates similarly in vivo remains to be determined [141,142]. Through this pathway, Schwann cells may establish a metabolically permissive environment for bone formation by regulating amino acid and lipid metabolism in preosteoblasts, though these findings require confirmation in more physiologically relevant models. However, the role of Schwann cells in in vivo heterotopic ossification remains an area of active investigation. Additionally, following nerve injury, Schwann cells undergo a phenotypic shift toward a repair-associated state marked by cytokine secretion, antigen presentation, and immune cell recruitment [143]. Although Schwann cells may not directly induce the pathological osteogenesis observed in tHO, they may function as context-dependent facilitators, up- or downregulating immune signaling and metabolic pathways.

For progenitors to exit the nerve, structural constraints must be transiently relaxed. The BNB is a dynamic, multicellular unit composed of endothelial cells, perineurial cells, Schwann cells, pericytes, and a basement membrane [50,62,144,145]. Cell–cell and cell–matrix interactions among axons, Schwann cells, macrophages, and mast cells regulate the BNB and control endoneurial permeability. Importantly, BMP2 has been shown to cross the BNB and activate endoneurial signaling without inducing nerve degeneration, indicating that BNB modulation during tHO reflects regulated permeability rather than barrier failure. Perineurial remodeling may also contribute to a tHO-permissive environment. Perineurial cells have been shown to undergo brown adipogenic differentiation and regulate matrix metallopeptidase-9 (MMP9), a critical mediator of BNB permeability during neurogenic tHO. While the exact mechanism remains incompletely understood, perineurial remodeling may represent an early step in the neuroinflammatory cascade, potentially allowing temporary loosening of the BNB and facilitating the migration of osteogenic progenitors from the peripheral nerve, though the mechanisms governing this process remain incompletely understood [144]. Importantly, this process appears to reflect regulated barrier plasticity rather than overt neurodegeneration, implicating the BNB as a potential site of maladaptation during chronic inflammation, a relationship that merits further investigation.

Within this permissive environment, endoneurial progenitors become mobilized. Progenitor populations expressing neural markers (PDGFRα, Musashi-1, p75^NTR) and endothelial-associated markers (Tie-2) have been identified within the endoneurial compartment and suggest a dual cell lineage in tHO [146,147]. These cells have been proposed as a key cellular source of ectopic osteoblasts, rapidly expressing osteogenic transcription factors following tHO induction and exiting the nerve via endoneurial vessels within 48 h, though the extent to which these findings translate to human tHO remains an open question. The expression of Claudin-5 during this process suggests that progenitor exit occurs through regulated vascular pathways rather than nonspecific barrier breakdown. However, the signals governing progenitor mobilization, selection, and differentiation remain largely undefined. Together, these findings illustrate the endoneurial compartment as a launch point for osteogenic misallocation in tHO. Once progenitors have exited the endoneurial compartment, immune cells may reinforce osteogenic differentiation. As mentioned previously, peripheral nerves contain resident macrophages that contribute to tissue homeostasis and nerve maintenance. Following nerve injury, degenerating axons and reactive Schwann cells recruit additional macrophages to the endoneurial compartment [148,149]. These recruited macrophages are phenotypically different from the resident macrophages and display heightened inflammatory activity. In tHO, sustained M1-polarized macrophage activation and osteoinductive cytokine secretion (TNF-α, IL-1β, IL-6) drive aberrant FAP differentiation, promoting ectopic bone formation [150]. Although evidence is emerging, recruited macrophages may act as pathological amplifiers during tHO through their involvement in mechanisms that stabilize osteogenic differentiation once neural- and progenitor-driven processes have been initiated.

## 6. Clinical Relevance of the Neuro-Immune Axis in tHO

Viewing tHO pathogenesis from the neural–immune axis opens various novel avenues for therapeutics with clinical relevance. Biomarkers for early detection of tHO prior to radiographic evidence are one such avenue. Emerging molecular studies have identified several novel regulators of tHO that may serve both as biomarkers and therapeutic targets. These biomarkers reflect dysregulated inflammatory and neuroimmune signaling, which are recognized precursors to tHO formation. Increasing evidence implicates noncoding RNAs (ncRNAs)—particularly microRNAs (miRNAs) and long noncoding RNAs—as sensitive early indicators capable of identifying tHO before overt tissue ossification occurs [151]. Transcriptomic analyses of tHO tissue demonstrate enrichment of genes associated with leukocyte activation and cytokine signaling compared with healthy muscle, consistent with sustained inflammatory responses to injury. Among ncRNAs, miR-148 has been reported to be significantly elevated in tHO lesions and is similarly increased in several chronic inflammatory conditions [151]. Additional studies have identified increased expression of miR-99b, miR-146, and miR-204 within tHO tissues. The miR-99 family is known to be expressed in hematopoietic stem cells, suggesting a potential role in immune-mediated processes [151]. Upregulation of miR-146 has been associated with enhanced fibroblast proliferation and amplified inflammatory signaling, processes that may contribute to the pathological remodeling seen in tHO [151,152]. Although the functional role of miR-204 remains incompletely defined, emerging data suggest it may serve as a useful biomarker for early tHO development [71]. LINC00320, a long noncoding RNA, inhibits Wnt/β-catenin signaling by binding to β-catenin, further affecting the osteogenic differentiation in MPCs, suggesting its importance in tHO formation and another early biomarker [152]. Collectively, these findings support the concept that dysregulated ncRNA expression profiles represent a promising biomarker platform for identifying tHO at a pre-radiographic stage and provide minimally invasive tools for early diagnosis and risk stratification [153]. In addition to ncRNA expression, other underappreciated risk factors for tHO, such as NINI and soft tissue infections, should also be considered. CGRP and SP are strongly upregulated in early tHO lesions and are also potential early biomarkers for tHO. TLR3 is a toll-like receptor responsible for detecting PAMPs and anti-viral response and has been detected in high amounts within tHO tissues. This possibly highlights the importance of infections as a biomarker to flag or target to prevent the progression of tHO within traumatic injuries [151,154].

Current tHO prophylaxis relies on empirical NSAIDs, bisphosphonates (etidronate) or low-dose radiation, which are empirical and do not directly target osteogenic or neuroimmune pathways. NSAIDs treat the initial stages of tHO by stopping inflammation from local signaling after tissue injury, such as IL-6 and TNF [155]. They show high efficacy when administered soon after the initial injury, but low efficacy once tHO has already formed [19]. Radiotherapy prevents the osteogenic differentiation of MNCs, preventing aberrant growths. It can be applied both before and after surgery is conducted, with little difference in efficacy between the two applications [19]. If radiotherapy is delayed after surgery, however, efficacy begins to decrease. Bisphosphonates are antiresorptive agents that inhibit osteoclast-mediated bone turnover and have been investigated for the prevention and treatment of tHO. Etidronate (disodium etidronate), a first-generation, non–nitrogen-containing bisphosphonate, acts primarily by incorporating into hydroxyapatite crystals and impairing their growth, thereby inhibiting mineralization of newly forming bone. In contrast to newer nitrogen-containing bisphosphonates that primarily suppress osteoclast activity, etidronate can directly interfere with ectopic calcification and osteoid maturation, making it historically attractive for tHO prophylaxis. Clinical studies have reported mixed efficacy: early administration following trauma or surgery may reduce radiographic progression of tHO, but etidronate does not reliably prevent the initial osteogenic differentiation of mesenchymal progenitor cells and may allow recurrence once therapy is discontinued. Moreover, prolonged use can impair normal skeletal mineralization, potentially leading to osteomalacia, which limits long-term dosing. Because tHO pathogenesis involves both osteogenic differentiation and subsequent mineralization, etidronate is likely targeting only downstream stages of lesion maturation rather than upstream inflammatory, neuroimmune, or BMP-driven processes. For this reason, contemporary management strategies favor NSAIDs and radiation for prophylaxis, while bisphosphonates are generally reserved for select cases or adjunctive use [156,157]. Thus, current therapies are ineffectual, have limiting side effects and lack spatial and temporal specificity in the complex pathogenesis of tHO, highlighting a crucial need for biomarkers for early identification and stratification of high-risk patients, as well as temporally precise therapeutics to mitigate tHO [158].

Increasing understanding of the molecular signal transduction pathways underlying tHO has revealed numerous candidate targets for precision therapy. Macrophage polarization is central to disease progression: pro-inflammatory M1 macrophages establish a permissive inflammatory microenvironment following injury, whereas M2 macrophages regulate BMP-2–driven osteogenic differentiation of mesenchymal stem cells [155]. Accordingly, immune-modulating approaches such as IL-1β inhibition or pharmacologic agents like metformin—which promotes M2 polarization—may attenuate tHO development [159]. As detailed in Section 2, SP-mediated neuroinflammation stimulates CGRP release and mast cell degranulation, and pharmacologic interruption of these neuropeptide axes represents a rational therapeutic strategy. Retinoic acid receptor-γ (RARγ) agonists represent another promising strategy, as retinoid signaling suppresses chondrogenic differentiation of progenitor cells [156]. The synthetic agonist palovarotene has demonstrated substantial efficacy by reducing inflammation and inhibiting BMP signaling and is now approved for hereditary tHO associated with fibrodysplasia ossificans progressive [156,158]. Because BMP signaling lies at the core of tHO pathogenesis, agents that disrupt this pathway—such as activin A, which promotes SMAD2/3 signaling and antagonizes BMP-SMAD1/5/8 activity—can inhibit osteogenic gene transcription [??]. Metabolic pathways also intersect with osteogenic signaling: the mammalian target of rapamycin (mTOR) pathway promotes chondrogenesis and osteogenesis via mTORC1, which is sensitive to rapamycin, while AMP-activated protein kinase (AMPK) activation—achieved with agents such as metformin—suppresses both mTORC1 and BMP/SMAD signaling, highlighting therapeutic crosstalk between metabolic and osteogenic pathways [160]. Additional anti-inflammatory strategies include inhibition of NF-κB, a transcription factor that drives osteogenesis in inflammatory contexts and can be suppressed by glucocorticoids such as prednisone or dexamethasone [161]. Combinations of thiazolidinediones, indomethacin, and dexamethasone have been shown to decrease substance P and BMP-2 levels [162]. Noncoding RNAs, previously identified as early biomarkers, may themselves serve as therapeutic targets or precision-medicine guides to tailor treatment selection [163].

In addition to immunomodulation, therapeutics targeting NINI are also of interest. The CGRP pathway already has FDA-approved medication, including monoclonal antibodies targeting the CGRP ligand (fremanezumab, galcanezumab, eptinezumab) or the CGRP receptor (erenumab), as well as small-molecule CGRP receptor antagonists (“gepants”) used for acute and preventive migraine therapy. For tHO, CGRP blockade is conceptually appealing because CGRP has documented roles in bone-repair biology and can drive osteogenic programs via cAMP/PKA/cAMP–cAMP-response element binding protein (CREB) signaling in stem/progenitor contexts—exactly the signaling space implicated in aberrant endochondral ossification after trauma. Translationally, two implementation strategies are worth emphasizing in a review: (i) systemic short-course prophylaxis in very high-risk injuries (e.g., polytrauma with burns/TBI, major orthopedic trauma with high inflammatory burden), and (ii) local delivery (hydrogel/depot formulations) at the injury/surgical site to improve spatial specificity and reduce systemic exposure—important because CGRP is a physiologic vasodilator, and long-term suppression in other indications has raised attention to vascular and gastrointestinal tolerability considerations in real-world use. Practically, a tHO trial design could enrich for patients with early molecular signatures (e.g., neuroimmune biomarkers, rising inflammatory cytokines) and use early endpoints such as pain/hyperalgesia trajectories, circulating neuropeptide levels, and MRI/ultrasound changes prior to radiographic ossification, alongside definitive outcomes (CT volume, functional ROM, reoperation) [37,164].

**Figure 3:**
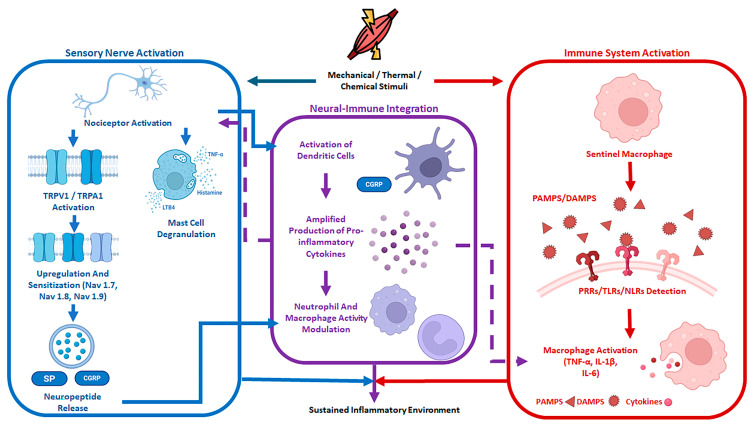
Neural–immune crosstalk in tHO. Schematic illustration of the bidirectional signaling between the sensory nervous system and the immune system that sustains the inflammatory environment driving tHO. Mechanical, thermal, and chemical stimuli from tissue injury simultaneously activate sensory nerves (left, blue) and the innate immune system (right, red), with integration occurring at the neural–immune interface (center, purple). Within the sensory nerve compartment, nociceptor activation triggers TRPV1/TRPA1 channel activation, upregulation and sensitization of Nav1.7, Nav1.8, and Nav1.9, and subsequent release of neuropeptides SP and CGRP. In parallel, mast cell degranulation releases histamine, LTB4, and TNF-α, which further amplify nociceptive signaling. Within the immune compartment, sentinel macrophages detect PAMPs and DAMPs via PRRs, TLRs, and NLRs, culminating in macrophage activation and release of pro-inflammatory cytokines TNF-α, IL-1β, and IL-6. At the neural–immune integration zone, CGRP drives activation of DCs, amplified production of pro-inflammatory cytokines, and modulation of neutrophil and macrophage activity, collectively sustaining the inflammatory environment that promotes osteogenic differentiation of progenitor cells and tHO.

As noted above, SP is strongly upregulated in early tHO lesions and across multiple preclinical models, and is positioned upstream of immune cell recruitment and mast cell activation (see Figure 3), processes repeatedly linked to tHO permissiveness. The most clinically available SP-pathway drugs are NK1 receptor antagonists (e.g., aprepitant and IV fosaprepitant, among others), which competitively block SP binding at NK1 receptors and are widely used as antiemetics in oncology settings. This creates an attractive repurposing path for tHO prophylaxis, especially for short, peri-traumatic dosing windows aimed at the earliest neurogenic inflammatory phase. However, the literature also argues for caution and nuance: in at least one murine FOP model evaluating mast-cell–targeted strategies, cromolyn reduced tHO whereas aprepitant did not, suggesting that NK1 antagonism may be insufficient alone (or may miss relevant NK1-independent mast-cell activation biology) in some contexts. A balanced review can therefore frame SP/NK1 blockade as a component of a multi-node strategy—potentially paired with mast-cell stabilization, macrophage modulation, or osteogenic-pathway inhibitors—to overcome pathway redundancy and context dependence [37,165]. Given the extensive redundancy among signaling pathways, combination therapies targeting multiple nodes are likely to be most effective in mitigating tHO and preventing recurrence. Importantly, tHO follows a predictable temporal sequence that necessitates stage-specific interventions [19,155]. This framework enables patient stratification into early versus late disease stages and underscores the need for precision, mechanism-based treatments to replace current empiric approaches. The pharmacologic targets discussed in this section are summarized in Table 1. While the therapeutic targets discussed above are promising, it is important to acknowledge that the majority of supporting evidence remains preclinical, derived primarily from murine models. Notable exceptions include palovarotene, which is FDA-approved for FOP, and the CGRP and NK1 antagonists, which are clinically approved for migraine and chemotherapy-induced nausea, respectively, but have not yet been evaluated in tHO-specific clinical trials. Key translational challenges shared across these candidates include the identification of optimal dosing windows relative to injury timing, the risk of off-target effects in critically injured patients, and the absence of validated biomarkers to stratify patients most likely to benefit. Addressing these challenges through well-designed translational and early-phase clinical studies will be essential before any of these agents can be recommended for routine tHO prophylaxis.

## 7. Conclusions

tHO is a significant clinical condition with a paucity of prognostic biomarkers, prophylaxis or therapeutic measures. Recent studies have implicated sensory neurons within injured tissues and the resultant NINI as important contributors to tHO initiation, though the full extent of their role in clinical disease continues to be investigated. The role of NICUs and the neural–immune axis in tHO is complex, highlighting their dual functions in tissue repair and aberrant bone formation. As our understanding of NIA in tHO continues to evolve, innovative therapies targeting macrophage functions and neural inflammation hold considerable promise, pending rigorous translational investigation. Immunomodulators, CGRP monoclonal inhibitors (such as fremanezumab), and drugs that regulate macrophage polarization have shown encouraging results in preclinical studies, and their clinical translation represents an exciting frontier that requires carefully designed trials. Single-cell and spatial transcriptomics will further help explore the neural–immune microenvironment and its interactions in tHO, providing new insights into both early biomarkers and precision prophylaxis for high-risk patients. Given the complex pathological process of tHO, multi-target and multi-level therapies may prove to be more effective. Finally, the use of preclinical in silico and in vivo models will guide the efficacy and safety of these novel therapeutic strategies in the future, offering more precise and effective treatment options for tHO patients.

## Figures and Tables

**Table 1:** Emerging Pharmacologic Targets in the Neural–Immune Axis for the Prevention and Treatment of tHO.

System Involved	Family of Drug	Individual Drug	Mechanism of Action	Evidence Level	References
Immune	Biguanide	Metformin	Promote M2 macrophage polarization	Preclinical (murine models); no tHO-specific clinical trials	Ren et al. [159]
Immune	RARγ agonists	Palovarotene	Inhibit BMP signaling	FDA-approved for FOP; investigational for tHO	Felix-Ilemhenbhio et al. [158]
Immune	mTOR inhibitor	Rapamycin	Inhibit mTORC1 pathway	Preclinical (murine models); no tHO-specific clinical trials	Wu J, et al. [160]
Immune	Glucocorticoids	Prednisone/dexamethasone	Inhibit NF-κB signaling	Clinical use for inflammation; not validated for tHO prevention	Sinha et al. [161]
Neural	CGRP ligand monoclonal antibodies	Fremanezumab, galcanezumab, eptinezumab	Bind circulating CGRP ligand to prevent CGRP receptor activation	FDA-approved for migraine; no tHO clinical trials to date	González-Hernández et al. [166]
Neural	CGRP receptor monoclonal antibodies	Erenumab	Binds CGRP receptor to inhibit CGRP receptor activation	FDA-approved for migraine; no tHO clinical trials to date	Bhakta et al. [167]
Neural	Small-molecule CGRP receptor antagonist	“-gepants”	Competitive antagonism of the CGRP receptor	FDA-approved for migraine; no tHO clinical trials to date	Dubowchik et al. [168]
Neural	NK1 receptor antagonists	Aprepitant, fosaprepitant	Block SP binding at NK1 receptors	FDA-approved for chemotherapy-induced nausea; preclinical evidence only for tHO	Navari et al. [169]

## Data Availability

This is a review article, and no new data were created.

## Abbreviations

The following abbreviations are used in this manuscript:

Abbreviation	Full Term
ACh	Acetylcholine
AMPK	AMP-activated protein kinase
BMP-2	Bone morphogenetic protein-2
BMPRI	BMP type I receptor
BMPRII	BMP type II receptor
BNB	Blood–nerve barrier
cAMP	Cyclic adenosine monophosphate
CGRP	Calcitonin gene-related peptide
CREB	cAMP-response element binding protein
CRLR	Calcitonin receptor-like receptor
DAMPs	Damage-associated molecular patterns
DCs	Dendritic cells
DRG	Dorsal root ganglion
FAP	Fibro-adipogenic progenitor
HIF-1α	Hypoxia-inducible factor 1-alpha
HIF-1β	Hypoxia-inducible factor 1-beta
HMGB1	High mobility group box 1
HNK1	Human Natural Killer-1
IL-1β	Interleukin-1 beta
IL-6	Interleukin-6
IL-17A	Interleukin-17A
IL-23	Interleukin-23
LINC00320	Long intergenic non-coding RNA 00320
LTB4	Leukotriene B4
MAPK	Mitogen-activated protein kinase
MIF	Macrophage migration inhibitory factor
miRNA	MicroRNA
MMP9	Matrix metalloproteinase-9
MPCs	Mesenchymal progenitor cells
MSC	Mesenchymal stem cell
mTOR	Mammalian target of rapamycin
mTORC1	mTOR complex 1
Nav	Voltage-gated sodium channel
NCDPCs	Neural crest-derived progenitor cells
ncRNA	Noncoding RNA
NF-κB	Nuclear factor kappa B
NGF	Nerve growth factor
NIA	Neural–immune axis
NICUs	Neuro-immune cell units
NINI	Nociception-induced neural inflammation
NK1	Neurokinin-1
NLRs	NOD-like receptors
NSAIDs	Non-steroidal anti-inflammatory drugs
OSM	Oncostatin M
Osx/Osterix	Osterix (osteogenic transcription factor)
p38 MAPK	p38 mitogen-activated protein kinase
PAMPs	Pathogen-associated molecular patterns
PDGFRα	Platelet-derived growth factor receptor alpha
PGE_2_	Prostaglandin E2
PKA	Protein kinase A
PKC	Protein kinase C
PRRs	Pattern recognition receptors
RAMP1	Receptor activity-modifying protein-1
RARγ	Retinoic acid receptor-gamma
Runx2	Runt-related transcription factor 2
SMAD1/5/8	Small mothers against decapentaplegic 1/5/8
SMAD4	Small mothers against decapentaplegic 4
SOX5/6/9	SRY-box transcription factors 5, 6, and 9
SP	Substance P
TAK1	TGF-β-activated kinase 1
TBI	Traumatic brain injury
TGF-β	Transforming growth factor-beta
TGFβR1	Transforming growth factor-beta receptor 1
tHO	Trauma-induced heterotopic ossification
TLRs	Toll-like receptors
TNF-α	Tumor necrosis factor-alpha
TrkA	Tropomyosin receptor kinase A
TRP	Transient receptor potential
TRPA1	Transient receptor potential ankyrin-1
TRPV1	Transient receptor potential vanilloid-1
VEGF	Vascular endothelial growth factor
